# A First Verification of Sim*2D*Sphere Model’s Ability to Predict the Spatiotemporal Variability of Parameters Characterizing Land Surface Interactions at Diverse European Ecosystems

**DOI:** 10.3390/s25051501

**Published:** 2025-02-28

**Authors:** Christina Lekka, George P. Petropoulos, Spyridon E. Detsikas

**Affiliations:** Department of Geography, Harokopio University of Athens, El. Venizelou St., 70, 17671 Athens, Greece; clekka@hua.gr (C.L.); sdetsikas@hua.gr (S.E.D.)

**Keywords:** earth observation, SVAT, Sim*2D*Sphere, land surface interactions, surface heterogeneity, FLUXNET

## Abstract

Land–atmosphere interactions (LSIs) involve intricate complex processes that drive critical exchanges of energy and matter that influence ecosystem and climate dynamics, with variations in ecosystem responses and feedback effects depending on their specific environmental characteristics. To this end, this study represents the first extensive validation of the Sim*2D*Sphere model, to establish its coherence and use in the study of LSIs across a range of biomes and climatic zones. For this purpose, water and energy fluxes from a total of 10 eddy covariance sites and a total of 12 calendar days were analyzed. Earth observation (EO) data were integrated with ground observations at the different sites to execute the Sim*2D*Sphere model. The diurnal dynamics of energy fluxes were compared against corresponding ground measurements. The results showed that the highest accuracy was observed for the grassland sites (R^2^ from >0.85; RMSE < 68.50 Wm^−2^), whereas the lowest accuracy was found in forest sites (R^2^ from >0.80; RMSE < 75.0 Wm^−2^). All in all, these initial results obtained herein are very promising and demonstrate the models’ promising potential in the study of LSIs at variant spatiotemporal resolutions.

## 1. Introduction

Land surface and atmosphere interactions (LSIs) are critical components of the Earth’s climate system, influencing weather patterns, hydrological cycles, and ecosystem dynamics [1,2]. For example, LSIs can have an important effect on atmospheric conditions, leading to variations in precipitation and temperature patterns [3,4,5]. Obtaining a better understanding of LSIs is essential for addressing global challenges such as climate change and food security [6,7]. Adequate knowledge of LSIs can also provide valuable information to stakeholders and decision-making centers, supporting evidence-based policies and adaptation strategies [8]. Among the most important LSI parameters are the latent heat flux (LE), sensible heat flux (H), and net radiation (R_n_), as LE and H are interrelated with fluctuations in air temperature through changes in the state of heat fluxes [9]. This is due to their key role in ecosystem formation by controlling interactions and feedbacks within the soil/vegetation/atmosphere continuum [10].

One of the most widely used approaches to measure those parameters includes the use of ground instrumentation, such as eddy covariance systems [11]. Those use fast-response sensors to capture variations in wind speed and scalar variables such as carbon dioxide and energy. Some of the key advantages of this approach include accurate estimates of water, energy, and carbon flux as well as of other micrometeorological parameters on regional and local scales. Yet, ground-based instrument networks often face challenges for large-scale use due to lack of spatial coverage and variability, complexity, and installation cost. As part of efforts for the monitoring of LSI of different ecosystems globally, ground observational networks have been developed. Among the most well-established networks is FLUXNET (https://fluxnet.org/ (accessed on 9 December 2024)) which provides invaluable data for climate modeling, ecosystem research, and the validation of remote sensing products [12]. FLUXNET datasets have also been used as a reference for benchmarking simulated outputs of ecological or meteorological models [13].

An alternative approach to studying LSIs involves the use of mathematical or physical simulation process models. Several such models have been developed for the study of energy and carbon dynamics at different geographical scales [14,15]. Among them, the most promising include land–biosphere models (LBMs), which range from simple land surface models (LSMs) to more complex and advanced systems that incorporate biogeochemical cycles. Those can accurately capture the variability of the water and carbon cycles [16,17,18].

A special category of LBMs includes the so-called Soil Vegetation Atmosphere Transfer (SVAT) models. Those are deterministic simulation models that describe the physical processes controlling energy and mass transport in the soil/vegetation/atmosphere system. SVAT schemes often combined with an Atmospheric Boundary Layer (ABL) model are commonly employed to produce essential simulations of energy, biochemical, and water parameters from field to regional scales. For example, ref. [19] used the ISBA and ISBA-MEB models to evaluate the energy budget of irrigated maize crops in Southwest France, suggesting the models accurately simulated evapotranspiration and soil moisture. For example, [20] compared ISBA, ORCHIDEE, and a diagnostic model for simulating energy, water, and vegetation interactions, concluding that ISBA and ORCHIDEE accurately simulate important ecological processes. Although those models provide often a detailed description of the physical processes that aim to simulate and function at time steps compatible with atmospheric process dynamics, challenges remain in their use due to their difficulty in capturing the natural processes at varying scales and architectural complexity levels, particularly so under highly heterogeneous environments [21,22,23].

To address some of the challenges associated with the use of LBMs, an advanced approach includes the use of Earth Observation (EO) datasets coupled with LBMs, and various data assimilation methods have been developed for this purpose [14,24]. This integration allows combining the fine temporal continuity and vertical coverage of such models, together with the horizontal coverage and spectral resolution of EO data; limits the propagation of uncertainty from input data to output data; and can support the parameterization processes [25]. It also addresses challenges related to sub-grid variability and the influence of spatial heterogeneity on carbon, water, and heat fluxes sources and sinks [26]. Integrating EO data significantly improves model simulations by providing a more accurate representation of vegetation and soil properties through detailed geospatial inputs, which reduces uncertainty in model input parameterization and in simulation outputs [27].

Among others, [28] have emphasized the importance of integrating EO data with SVAT models to improve estimates of ecosystem energy, carbon, and water fluxes. A variety of different approaches have been proposed to perform this synergy between EO and SVAT models. For example, [29] integrated thermal infrared EO data with an SVAT model to access the variability of evapotranspiration across an aridity gradient in Australia. This study findings supported that SVAT models integrated with EO data could capture successfully the spatial variability of evapotranspiration. In another study, [30] integrated EO data in the parameterization process of the LWF-Brook90 SVAT model, including vegetation indices and soil moisture information, for simulating water fluxes, soil moisture, and drought stress that improved the model’s representation of hydrological processes and its ability to simulate drought stress across ecosystems. Similarly, [31] used optical EO data to parameterize an SVAT model for soil and plant interactions and water availability in Swiss forests during the 2015 and 2018 droughts. The input EO data, including vegetation indices and canopy characteristics derived from MODIS, provided essential inputs that improved the models’ ability to capture the impact of droughts on the forest ecosystems and display forest water dynamics. More recently, [32] used EO data for the parameterization of the JULES model for various access land cover types in the tropical Andes. In this research study, EO data provided inputs, such as land cover types, vegetation indices, and surface properties, that improved the model’s representation of land–atmosphere interactions and refined its ability to spatially capture the region’s hydrological and ecological dynamics.

SimSphere SVAT model has presented important advancements in the recent years, particularly in recent years, with its use rapidly expanding globally where the model is used either as a stand-alone tool or synergistically with EO datasets. Beyond its use as an independent modeling tool, SimSphere has recently been modified to incorporate EO data for simulations at multiple spatial scales corresponding to the resolution of the EO datasets, effectively capturing spatial heterogeneity and variability [21,22,33]. A new add-on has been recently added to SimSphere, named Sim*2D*Sphere, which allows the integration of EO data in the parameterization process [25]. Sim*2D*Sphere is now able, for the first time, to accommodate as input data the observations that come from EO sensors, which acquire spectral data at different parts of electromagnetic radiation (EMR). With this advancement, Sim*2D*Sphere introduces, the capability to incorporate land surface heterogeneity into the model’s outputs, considering variations in both land cover and soil types. Initial validations of the modified Sim*2D*Sphere toolkit have shown promising results in accurately simulating energy fluxes at a regional scale [25]. Yet, a more comprehensive verification of Sim*2D*Sphere outputs relevant to LSIs retrievals is required to assess its performance across different soil and PFTs, as well as geographical zones.

In the purview of the above, the objective of this study is two-fold: (a) to assess the accuracy of Sim*2D*Sphere in capturing the diurnal local variations and to produce explicit spatial maps of instantaneous energy and heat fluxes, across different geographical zones, and (b) to determine Sim*2D*Sphere’s effectiveness to simulate LSIs across diverse Plant Functional Types (PFTs) based on EO-based geospatial data. An innovative aspect of this study is the demonstration of the synergistic use of Sim*2D*Sphere with EO data acquired from sensors operating in the visible, near-infrared, and thermal infrared domains of the EMR to provide estimates of the key parameters characterizing land surface interactions. To assess the model predictions, the model outputs were compared for selected days against local-scale in-situ observations derived from 10 European FLUXNET experimental sites.

## 2. Experimental Set-Up

### 2.1. Sites Description

**Figure 1:**
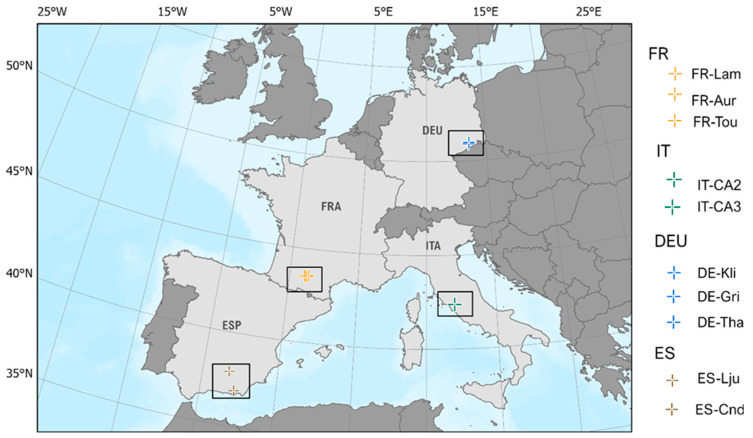
Eddy Covariance sites from the FLUXNET2015 database (https://fluxnet.org/data/download-data/ (accessed on 9 October 2024)) and ICOS portal (https://data.icos-cp.eu/portal/ (accessed on 9 October 2024)) across Europe, used for the validation of the model’s simulations.

To evaluate the Sim*2D*Sphere output products, a total of ten eddy covariance (EC) sites across Europe were selected based on several pre-defined criteria. The criteria for their selection were the following: (a) each site needed to represent various land cover types to evaluate the model’s effectiveness in simulating fluxes across different land uses; (b) sites had to display consistent land cover, uniform topography, and minimal human influence; and (c) data from each site required simultaneous measurements of the five validated parameters for the same date. Finally, (d) the energy balance closure (EBC) was computed for each of the potential days and those with a low or imbalanced Energy Balance Ratio (EBR) were removed or replaced (with other days), indicating a discrepancy between the measured incoming and outgoing energy (Mauder et al. [34]). To this end, only sites that met these criteria were included in the analysis. The characteristics of the experimental sites that were used in this research are outlined in Table 1. The selected sites represented various ecosystems, each displaying different characteristics to incorporate a variety of conditions in the model evaluation. These sites represent six distinct biomes, including forest (i.e., deciduous broadleaf forest and evergreen needleleaf forest), cropland, grassland, open shrubland, and woodland savanna (Figure 1). Considering spring, summer, and early fall seasons across different years for half-hourly data (winter months excluded), a comprehensive dataset for model tuning and validation was collected (Table 1).

### 2.2. Datasets

#### 2.2.1. FLUXNET Ground Monitoring Network

To evaluate the Sim*2D*Sphere model predictions, the European ecosystem sites were selected from the FLUXNET2015 network of micrometeorological towers and the Integrated Carbon Observations System (ICOS). At each site, a set of micrometeorological parameters was acquired, including the turbulent fluxes of latent heat (LE) and sensible heat (H), and net radiation (R_n_) at the surface. For detailed information about FLUXNET datasets and the processing of raw data, the reader can refer to [35,36].

#### 2.2.2. EO and Geospatial Data

The study incorporated several geospatial datasets for the model’s parameterization accessed through the Google Earth Engine (GEE) platform. Landsat-8 satellite imagery at a 30 m resolution was used for the parameterization of vegetation components. Additionally, the 90 m Shuttle Radar Topography Mission’s Digital Elevation Model (SRTM DEM) provided the necessary elevation data for the parameterization of topographical features. Since the SRTM DEM corresponds to data acquired in previous years (2000s), temporal differences between the datasets are assumed to have minimal effect given the relatively stable nature of topographical features over time (i.e., as no significant changes in the DEM are expected due to the time gap between the datasets). The geospatial product of Corine Land Cover (2018) was used to access the spatial surface heterogeneity and to incorporate different land cover classes into the model’s parameterization.

#### 2.2.3. Radiosondes Data

Meteorological atmospheric profile data obtained from a publicly available weather balloon data archive. These radiosondes are available within the University of Wyoming Atmospheric Science Radiosonde Archive (https://weather.uwyo.edu/upperair/bufrraob.shtml (accesed on 3 September 2024)).The nearest stations to the selected experimental FLUXNET site provided local profiles of relative humidity, atmospheric pressure, temperature and dew point temperature, wind direction and wind speed. 

### 2.3. Sim2DSphere Model

Briefly, the SimSphere SVAT model employs a composite soil/vegetation/atmosphere continuum to simulate land surface energy exchanges across heterogeneous landscapes, leveraging the advanced capability of 2D spatialization to assess feedback processes within the Earth system. The configuration allows one to calculate important variables, including surface energy fluxes, soil moisture, and carbon dioxide exchanges, across user-defined time steps within a 24 h cycle. The solar radiation transfer mechanism is implemented through a multi-layer structure, displaying both sunlit and shaded conditions. The model provides a surface layer and a root zone, allowing for a complex representation of water and heat transfer dynamics. The model operates using a resistance analogy, like Ohm’s Law, to represent the complex interactions between soil and vegetation [37]. Stomatal resistance is parameterized to reflect the physiological responses of plants to environmental conditions, such as soil moisture and atmospheric carbon concentration [38].

A comprehensive reference to the SimSphere principles can be found in [38,39,40]. The capability of SimSphere SVAT model to simulate key parameters characterizing LSIs across various ecosystems research has been extensively demonstrated [33,41,42] and a series of sensitivity analyses have been performed confirming the architectural coherence of the model [43,44]. The Sim*2D*Sphere model, an advanced extension of the SimSphere SVAT model which was recently introduced, improves the representation of LSIs by incorporating the spatial dimensions into its diurnal simulations [25]. For detailed insights into the principles and the functional framework of the recently developed Sim*2D*Sphere module, readers can refer to the recently published work in [25]. The Sim*2D*Sphere model presents the focus of the present study, with particular emphasis on evaluating its outputs.

## 3. Methodology

**Figure 2:**
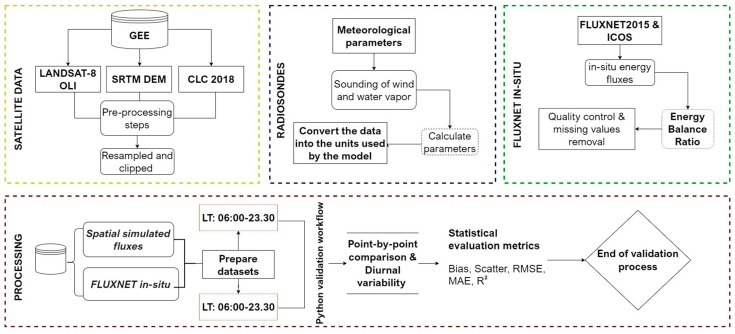
Overall methodological framework of the study.

To support reproducibility in handling datasets from diverse sources for meteorological modeling efforts, the processes were automated using a workflow developed in Python. This workflow was designed to streamline the pre-processing of multiple data types for use in the Sim*2D*Sphere SVAT model and to automate the validation process of in-situ measurements against simulated values through point-to-point comparisons of energy flux and temperature. The overall methodological framework is presented below (Figure 2).

### 3.1. Pre-Processing

The pre-processing of meteorological and geospatial data was performed using automated processes in Python 3.11.3. Specific pre-processing steps related to each dataset are briefly described in the corresponding sections below.

#### 3.1.1. FLUXNET In-Situ

Datasets from the FLUXNET (https://fluxnet.org/data/download-data/, accessed on 27 December 2024) and ICOS (https://www.icos-cp.eu/) databases were acquired in Level 2 of processing to ensure consistency and compatibility. A standardized approach to measure fluxes and perform calculations is implemented across all FLUXNET sites. This processing level included in-situ measurements, with instrumentation errors corrected or removed, and quality control and standard error correction procedures applied. The quality control and instrumentation error correction procedures for the in-situ measurements have already been carried out by the FLUXNET team, as a part of the standardized processing applied to FLUXNET data. Although EC is considered to be one of the most accurate methods for LE and H measurements, it has been generally documented that EC is obtained with an approximate 10 to 20% degree of uncertainty (Petropoulos et al., 2014 [45]) with a potential increase in uncertainty influenced by the topographical features of the site where the instrumentation is installed (i.e., hilly terrain) [45]. A detailed description of the uncertainties associated with the in-situ measurements can be found in FLUXNET documentation [35].

For the selected days with cloud cover less than 10% (according to optical satellite data), the energy balance closure (EBC) was calculated in order to assess the closure value of the EC measurements of turbulent fluxes. To calculate the EBC, the dataset was filtered to retain only those entries recorded between 00:00 and 23:30 h on the specified date.

The evaluation of EBC was mainly conducted through regression analysis [12,34]. The regression coefficients, including slope and intercept, as well as the coefficient of determination (R^2^), were derived from the ordinary least squares (OLS) technique, which examined the relationship between 30 min estimates of the dependent flux variables and the independently calculated available energy. The imbalance in energy closure is defined as the disparity between heat fluxes (LE + H) and net available radiation (R_n_ − G) based on the following formula:

(1) $$EBR=\sum \frac{LE+H}{R_{net}-G}$$

where *R_net_* represents net radiation, *G* represents the soil heat flux, *LE* is the latent heat flux, and *H* is the sensible heat flux.

The index typically ranges from 0 to 1, with values closer to 1 indicating relatively good energy closure over the diurnal cycle. Days with values close to 0 were either excluded from the analysis or replaced with alternative dates within the range of the summer season.

#### 3.1.2. Radiosondes In-Situ

The meteorological atmospheric data were used to parametrize the wind and water vapor profiles of the SVAT model at some level above the canopy. Radiosonde measurements include wind speed and direction, as well as temperature and dew point temperature at pre-defined geopotential heights and pressure levels at 06:00 GMT. Subsequently, numeric conversions were applied, converting geopotential heights to feet and wind speeds to knots, ensuring compliance with the modeling input requirements. Additionally, the temperature-dew point difference (T − Td) was calculated as required for model input.

#### 3.1.3. Geospatial Data

The Corine Land Cover (2018) dataset was used to adjust the plant types within the model’s vegetation group. For generic categorization, the Level-3 classification of CLC18 was applied, and specific plant types were then parameterized during the model’s set-up process.

Landsat-8 satellite EO data of 30 m spatial resolution, acquired on the GEE platform and processed as Collection 2 with atmospherically corrected surface reflectance, were inspected and filtered based on cloud and cirrus coverage. Initially, cloudy days with cloud cover greater than 5% were identified for each site within the defined time frame and excluded from further analysis. In the pre-processing phase, water bodies, clouds, and cloud shadows were masked based on the QA layer. In addition, topographical factors, such as slope and aspect, were produced from 30 m resolution SRTM’s digital elevation model (DEM) product.

The EO optical data were then used to generate vegetation parameters for the model’s parameterization. The fractional vegetation cover (FVC) was computed according to [46] using the NDVI relationship as shown in Equation (2).

(2) $$\mathrm{FVC}={\left(\frac{NDVI-{NDVI}_{min}}{{NDVI}_{max}-{NDVI}_{min}}\right)}^{2}$$

where *NDVI_min_* and *NDVI_max_* represent the NDVI values for bare soil and full vegetation cover, respectively.

Similarly, the Leaf Area Index (LAI), which quantifies the total leaf area per unit ground area and is an important indicator of vegetation density, is calculated from NDVI using the formula presented in Equation (3) [47,48].

(3) $$LAI=0.57\times exp\left(2.33\times NDVI\right)$$

The geospatial data were pre-processed to maintain consistency and relevance for the subsequent analyses. Initial steps included clipping raster datasets to the region of interest and excluding water bodies and urban areas. Given that the datasets originated from different sources, particularly the 500 m resolution CLC 2018 geospatial product, all the datasets were resampled to a consistent 30 m resolution. Finally, a combined binary mask was created to identify invalid NaN pixels, corresponding to excluded land cover classes as well as cirrus and clouds, across all the geospatial products, which were excluded from the simulation analysis.

### 3.2. Sim2DSphere Parameterization

Previous sensitivity analysis (SA) studies have identified the model input parameters that most significantly affect the model’s performance in simulating key parameters characterizing LSIs [49]. For those inputs, wherever possible, EO data in GeoTIFF format were used as inputs to set the model’s parameterization. For the remaining model inputs such as plant and soil physiological properties and detailed data on vegetation dynamics and soil composition, the model was adjusted using supplementary information or empirical values from the scientific literature.

A wide range of parameterization variables was gathered, including standard meteorological data (wind speed, temperature, and precipitation) and vegetation features (height, foliage albedo, and emissivity) along with plant’s physiological properties (leaf width, minimum stomatal resistance (s·m^−1^), cuticle resistance (s·m^−1^), critical leaf water potential (bar), critical solar parameter (Wm^−2^), and stem resistance (s·m^−1^)). Soil parameters (mean temperature, water availability, surface roughness, thermal inertia, and ground albedo and emissivity) and measurements of soil’s properties (saturated thermal conductivity, saturated volumetric water content, saturated water potential) were also adjusted during the parameterization process. For a detailed overview of the SimSphere SVAT model input parameters, the reader can refer to [41,50,51].

Overall, the initial and ancillary meteorological parameters were sourced from the half-hourly FLUXNET2015 archive and BADM (Biological, Ancillary, Disturbance, and Metadata) of FLUXNET and ICOS network (e.g., soil water content); standard literature, e.g., [48,49,50,51,52,53]; and available geospatial datasets. To maintain the integrity of the analysis, all the datasets were subjected to quality control to confirm the completeness and validity of both the FLUXNET2015 and radiosonde data along with the availability of geospatial data. The selected parameters from the FLUXNET2015 archive, along with other meteorological parameters from radiosondes, were collected corresponding to the target date and the initialization time of 06:00 LT.

Initially, the model was parameterized according to the specific conditions of each site, incorporating relevant information on soil and vegetation properties such as canopy height, soil moisture availability, foliage albedo, precipitable water, surface temperature, and surface roughness, along with additional variables, including wind and vapor profiles, and station height. In order to perform the simultaneous simulation of energy flux exchange across different PFTs, an average value of the parameterization for each site within a block of sites was calculated. Consequently, each site within a block received the mean value for input parameterization values that were not represented as geospatial products (i.e., the above-mentioned parameters). To this end, an average value of these parameters was set across all the sites for each simulation, representing all the biomes of the sites that are included within a block.

### 3.3. Statistical Comparisons

The performance of the Sim*2D*Sphere model in simulating the targeted variables was evaluated using the statistical metrics included in Table 2 below to provide a complete assessment of the model’s performance. Those metrics are commonly employed to validate the simulated outputs of LSIs against observational network measurements [19,25,29,54,55,56].

## 4. Results

The model predictions were compared with in-situ measurements from the FLUXNET2015 dataset and ICOS archive to validate the spatiotemporal diurnal variability of energy and water fluxes at half-hour intervals. The accuracy of flux simulations from Sim*2D*Sphere was assessed across ten different geographical zones of Europe, covering six different PFTs. Each site corresponds to a specific biome, featuring different plant species associated with that ecosystem type. The sites included in the analysis are associated with crops, forests, grass, shrubs, and woodland savanna biomes across various geographical zones to provide a thorough validation of Sim*2D*Sphere.

**Figure 3:**
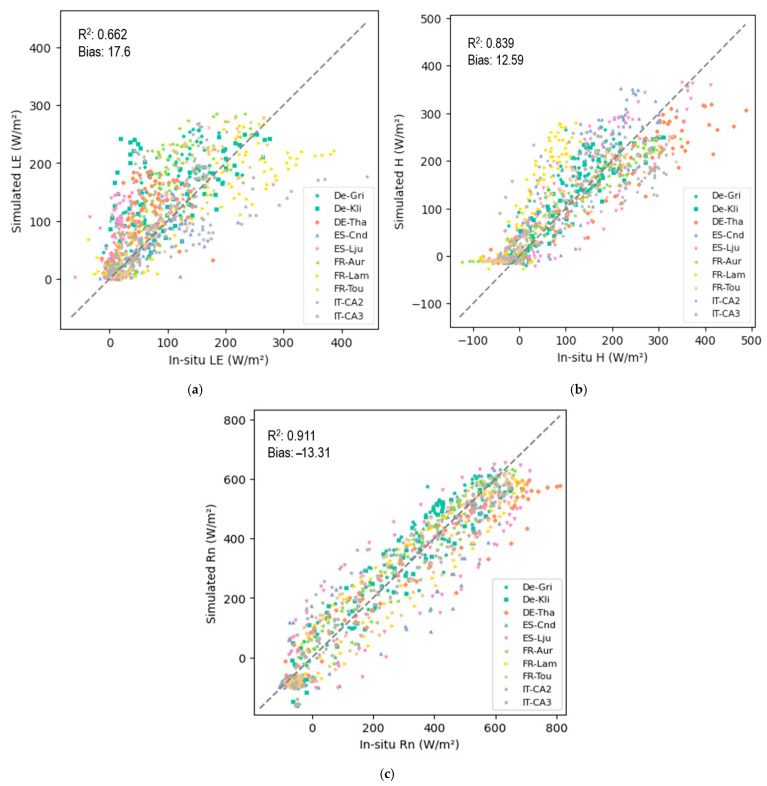
Scatterplot comparison of Sim*2D*Sphere-simulated and in-situ measurements across all stations, showing (**a**) LE flux, (**b**) H flux, (**c**) R_n_ flux, with a 1:1 reference line in each plot Bias is expressed in units of Wm^−2^.

The statistical results of the average performance for each flux across all the days and sites are presented in Table 3. A scatterplot comparison with a 1:1 reference line of the SimSphere-simulated and in-situ measurements for each parameter also across all the days and sites is provided in Figure 3. The performance of each flux under site-specific conditions and biomes is further discussed in the following sections.

**Figure 4:**
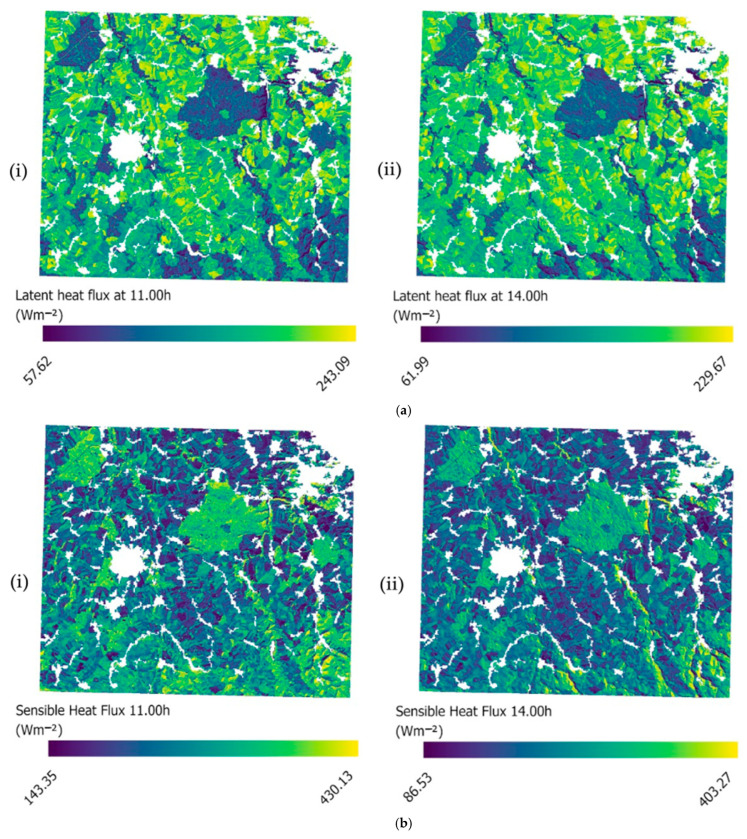

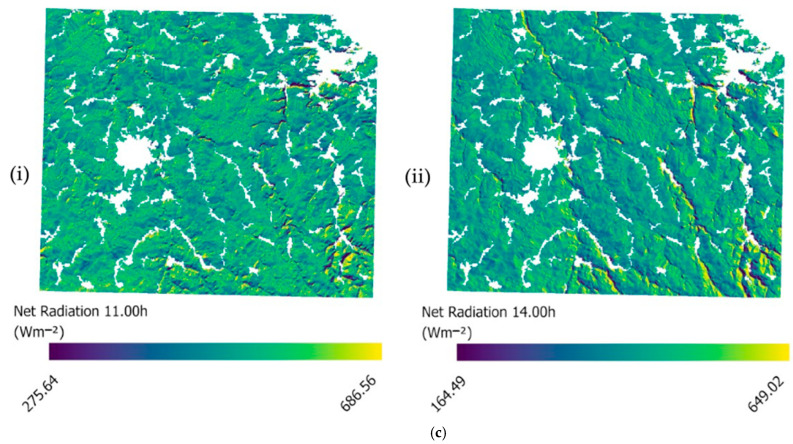
Diurnal variability of spatially explicit maps of the block of sites in Germany, which include three stations of grassland (DE-Gri), cropland (DE-Kli), and forest (DE-Tha) biomes. Examples of (**a**) LE, (**b**) H, and (**c**) R_n_ flux distribution at different times of day, i.e., at (i) 11:00 h and (ii) 14:00 h.

Comparisons were conducted across all the sites, simulated days, and biomes, with the associated average errors quantified and the results from the computation of all the statistical metrics also summarized. Examples of the spatiotemporal variability of the spatially explicit maps of heat and energy flux at two timestamps (11:00 h and 14:00 h) in Germany are presented in Figure 4. Each EC station (DE-Gri, DE-Kli, and DE-Tha) located in this block of sites represents a unique biome, allowing for the simultaneous analysis of diurnal energy fluxes associated with varying vegetation dynamics within a European context. The presented timestamps (i.e., 11:00 a.m. and 2:00 p.m.) were selected to be presented herein as they correspond closely to the overpass times of the Landsat satellite and similar polar-orbiting satellites.

**Figure 5:**
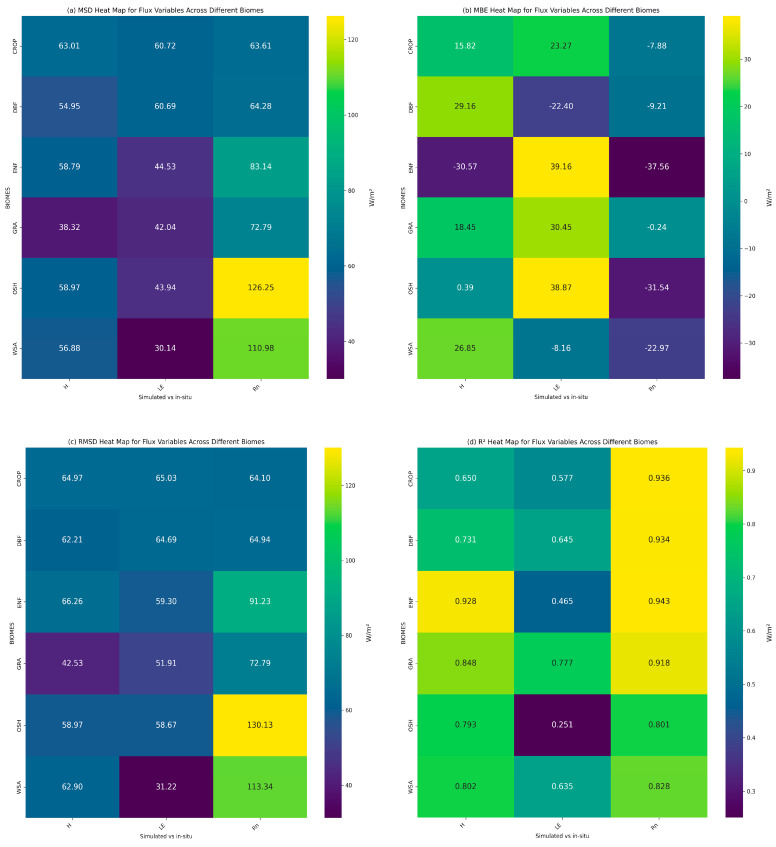
Heat maps showing the (**a**) Mean Squared Difference (MSD), (**b**) Mean Bias Error (MBE), (**c**) Root Mean Squared Difference (RMSD), and (**d**) R-squared (R^2^) values between the simulated and observed flux variables across different biomes. Each cell displays the statistic (Wm^−2^) for a specific flux type in each biome. The biomes include cropland (CROP), deciduous broadleaf forest (DBF), evergreen needleleaf forest (ENF), mixed forest (BIOMES), grassland (GRA), open shrubland (OSH), and woodland savanna (WSA). The energy flux variables of LE, H, and R_n_ are arranged on the *x*-axis. Higher values (in yellow and green) represent larger discrepancies between the simulated and observed values, while lower values (in purple and blue) indicate closer agreement. The color bar on the right shows the value of each statistic in Wm^−2^.

The averaged errors and agreement of the LE, H, and R_n_ parameters across all the days and sites for each biome are also provided in Figure 5. In terms of biome, as can be observed, higher accuracies across all the dates were achieved for the cropland and grassland sites. Overall, the simulations conducted for selected days during the summer season (June to August) showed improved performance compared to the simulations of the early autumn season (September), with higher agreement of the predicted and in-situ data for most of the parameters under investigation across all the sites and biomes. Specifically, overall simulations for France and Spain resulted in lower RMSD values and higher correlations in June and July, respectively. For Germany and Italy, simulations in August performed slightly better.

### 4.1. Latent Heat Flux

**Figure 6:**
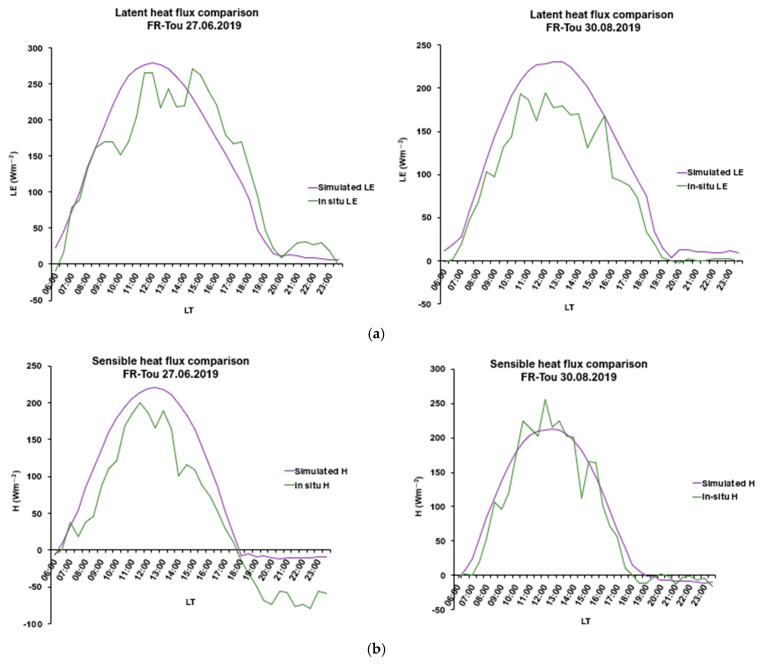

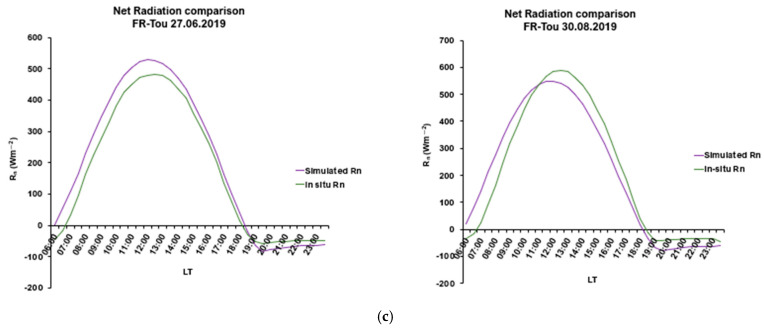
Comparison between in-situ FLUXNET and Sim*2D*Sphere for (**a**) LE, (**b**) H, and (**c**) R_n_, with diurnal simulations at 30 min intervals from 06:00 a.m. to 11:30 p.m. local time. The data corresponds to a grassland site within a block of sites in France (FR-Tou): for a day in late June (27 June 2019) and for a day in late August (30 August 2019).

For LE flux, the site-specific averaged RMSD varied from 29.30 to 66.16 Wm^−2^, indicating the model’s high potential to capture spatial variability across different biomes (Table 3). An average R^2^ value of 0.662 across all the sites suggested that the model performed with acceptable accuracy. Overall, when considering all the days and sites, the model’s estimates tended to result in a slight overestimation of the observed fluxes, as reflected by an average bias of 17.6 Wm^−2^ (Figure 3). Across all the days simulated, the model demonstrated low variance (MSD = 44.53 Wm^−2^), suggesting an accurate representation of LE amplitude. An example of diurnal variability in LE flux for a grassland site in France with a 30 min interval is shown (Figure 6a). As can be observed, the diurnal variability for each day in June and August was effectively captured, at least for the specific simulation dates. It is evident that the model was able to capture the diurnal variability of the observed LE fluxes, for the days of comparison.

With respect to crop type, the model’s highest averaged prediction accuracy for LE was observed at grassland and cropland sites (Figure 5). The sites of DE-Kli and FR-Lam both feature maize (C4), while FR-Aur has wheat (C3), and IT-CA2 is dominated by crop rotation system grassland. Sites of grassland biome demonstrated high prediction accuracy in both the southern and northern geographical regions (e.g., FR-Tou in France and DE-Gri in Germany), with the highest R^2^ value of 0.82 in FR-Tou across all the biomes in the block of sites in France. On a site-by-site basis, the cropland site of maize in Klingenberg yielded the highest accuracy (DE-Kli average RMSD of 29.30 Wm^−2^ and MBE = 7.53 Wm^−2^), with all the sites of cropland reaching relatively similarly levels of accuracy (Table 3). Overall, the DE-Kli achieved the highest R^2^ value of 0.89, followed by the same crop type in FR-Lam R^2^ of 0.80 (overall RMSD of 56.67 Wm^−2^, MBE = 38.427 Wm^−2^). IT-CA3 and FR-Aur crop sites (average RMSD of 64.68 Wm^−2^, MBE = −22.39 Wm^−2^ and RMSD of 66.16 Wm^−2^, MBE = 44.14 Wm^−2^, respectively) displayed a systematic underestimation and overestimation of LE, respectively, though both performed satisfactorily. The two forest types (ENF and DBF) cover distinct geographical areas, with DBF located south in Italy, (IT-CA2) and ENF located northern, in Germany (DE-Tha). The forests exhibited similar prediction accuracy for LE, with the ENF (DE-Tha) showing an RMSD of 59.30 and the DBF (IT-CA3) an RMSD of 64.68 and corresponding R^2^ values of 0.46 and 0.64, respectively. All in all, DBF and ENF across all the sites and days displayed acceptable RMSD values and low variance (Table 3). The WSA (ES-Cnd) and OSH (ES-Lju) sites located in Spain feature olive cultivars and evergreen shrubs (macchia), respectively. Generally, WSA outperformed OSH (averaged RMSD of 31.22 and 58.66 Wm^−2^, respectively). Additionally, R^2^ values support the better performance of WSA compared to OSH which obtained very poor (average R^2^ values of 0.63 and 0.25, respectively).

### 4.2. Sensible Heat Flux

According to Table 3, site-specific averaged RMSD for H flux from all the days varied from 38.57 to 99.03 Wm^−2^, reflecting a certain degree of uncertainty in accurately capturing spatial diurnal variations across the different biomes included. However, an average R^2^ value of 0.839 across all the sites and days suggests that the model’s predictions are acceptable (Table 3). Overall, the model’s estimates tended to slightly overestimate the observed fluxes, though they were predicted with satisfactory accuracy for most of the days in most of the sites, as reflected by a low average MBE of 12.59 Wm^−2^. Throughout all the simulated days, the model exhibited low variance (averaged MSD = 49.94 Wm^−2^) (Figure 3), indicating an effective representation of the H amplitude (Figure 4). An example of diurnal variability in H flux with a 30 min interval for a grassland site in France is presented in Figure 6b. As in the case of LE, the diurnal variability for each day in June and August was also successfully captured, especially for the day of August (Figure 6a). An overall underestimation of H flux is evident throughout the day. In contrast, the simulation results for August show a slight overestimation during the early noon hours, with the model clearly capturing the spatial pattern of H flux variability.

H flux achieved the highest prediction accuracy for maize and wheat crop sites (DE-Kli and FR-Aur). The model’s performance in grassland biomes was comparable to that of the crop sites. Promising statistical results were also observed in the grassland sites (FR-Tou and DE-Gri), with averaged R^2^ values of 0.88 and 0.86, and RMSD values of 41.14 and 43.86 W/m^2^, respectively (Figure 5). Across all the crop biomes, the H flux simulations generally produced low averaged RMSD values and low scatter. However, notable intra-site variability was observed across sites; e.g., for the maize cropland in France (FR-Lam), accuracy was rather moderate, inducing a large bias (average bias of 79.43). At the FR-Lam crop site, the high RMSD value (averaged RMSD = 99.03 Wm^−2^) was attributed to random variability rather than consistent bias. In contrast, other crop sites in the wider region, such as FR-Aur (average RMSD = 38.57 Wm^−2^), demonstrated lower averaged RMSD values, which underscores the site-specific nature of these deviations and suggests that localized factors may be driving the higher scatter observed at the FR-Lam site. The simulation of H flux across the forest sites in the analysis showed similar performance for both ENF and DBF, indicating an underestimation (MSD = −30.57 Wm^−2^) and overestimation (MSD = 29.16 Wm^−2^) of the H flux, with average RMSD values of 62.20 and 66.26 Wm^−2^, respectively. In general, the forest sites exhibited moderate to high prediction accuracy for H flux (Figure 5). Lastly, moderate results were obtained for the open shrublands and savannas regions in the block of sites in Spain, which correspond to averaged RMSD values of 58.96 Wm^−2^ and 62.90 Wm^−2^, respectively. The simulations of H flux displayed seasonal performance patterns similar to those of LE but presented considerably higher regional variability in RMSD. Notably, regional differences were also observed within a block of sites, for example, averaged RMSD ranged from 80.70 to 119.20 Wm^−2^ at the FR-Lam crop site in France on test days compared to a range of 22.30 to 49.53 Wm^−2^ at the FR-Aur crop site.

### 4.3. Net Radiation

The statistical analysis of the simulated R_n_ of all the sites provided an RMSD ranging from 48.73 to 140.23 Wm^−2^, with an average error of 80.66 Wm^−2^, indicating slight limitations in accurately capturing the diurnal variations in R_n_ flux across the different biomes, especially during days with inconsistent flux trends. An average R^2^ value of 0.911 across all the biomes suggests that the model’s predictions for clear-sky days without unusual flux variations are satisfactory (Table 3). Overall, the model tended to slightly overestimate observed fluxes, as reflected by a mean across all the sites of MBE = −13.31 Wm^−2^ (Figure 3) with an averaged RMSD of 80.76 Wm^−2^. Throughout all the simulated days, the model exhibited low variability and demonstrated consistent performance (MSD = 77.71 Wm^−2^), highlighting its potential to capture the R_n_ amplitude under different biomes (Figure 4). An example of diurnal variability of R_n_ at different time stamps within the 30 min interval for a grassland site in France is presented in Figure 6c. The model simulation closely corresponds to the observed R_n_ trend for the day in June with a minor underestimation in the early morning hours. For the remainder of the day, the predicted values correspond well to the observed values. On the day of August, the model initially underestimates R_n_ from morning to early noon, followed by a slight overestimation during the latter part of the day (Figure 6c). Despite these variations, the model successfully captures the overall spatial pattern of R_n_ variability, reflecting its ability to simulate the general fluctuations in net radiation across the day.

In terms of the diurnal comparisons, crop sites and grasslands showed the highest prediction accuracy across the biomes (Figure 5). However, model performance for the R_n_ parameter exhibited significantly higher error ranges across most statistical metrics in both the crop- and grassland biomes. On a per biome basis, the simulation of R_n_ across the sites exhibited varying levels of accuracy. For example, the cropland sites in France demonstrated high prediction accuracy, with FR-Aur showing an averaged RMSD of 48.74 Wm^−2^ (R^2^ = 0.964) and FR-Lam an averaged RMSD of 79.06 Wm^−2^ (R^2^ = 0.929), exhibiting close agreement between the simulated and observed fluxes. Similarly, the cropland and grassland regions in the block of sites in Germany (DE-Kli and DE-Gri), exhibited good prediction accuracy, with averaged RMSD values of 65.75 Wm^−2^ and 69.65 Wm^−2^, and R^2^ values of 0.93 and 0.93, respectively. The block of sites in Italy, corresponding to a forested (IT-CA3) and a cropland region (IT-CA2), yielded comparable moderate results, with averaged RMSD values of 58.94 Wm^−2^ and 64.94 Ε, and R^2^ values of 0.937 and 0.934, respectively. However, the model’s performance was variable at certain sites. For example, the performance of simulations at the OSH (ES-Lju) and SAV (ES-Cnd) sites of block in Spain was the poorest among all the sites, with averaged RMSD values of 140.23 Wm^−2^ and 113.33 Wm^−2^, respectively, indicating large discrepancies between simulated and observed fluxes (R^2^ values of 0.788 and 0.833). Another example is the forested site in higher latitudes, the ENF (DE-Tha) in the block of sites in Germany, where the averaged RMSD value was 91.23 Wm^−2^, suggesting a degree of inconsistency in R_n_ simulations. These discrepancies may have been induced by the parameterization approach as a result of using an average value within the block of sites (where sites within one satellite image were treated as a block) instead of a site-specific calibration of parameters, such as albedo and emissivity. This approach might not fully account for the distinct characteristics of forests compared to the other land cover classes, which can vary significantly in terms of their radiative and biophysical properties.

## 5. Discussion

### 5.1. Diurnal Dynamics of Energy and Radiative Fluxes

The model’s performance was tested across a wide range of environmental conditions and biomes, including all the selected days with flat and rugged terrain (max. slope less than <10%). This approach allowed for a comprehensive examination of Sim*2D*Sphere’s predictive ability even under non-ideal conditions. Notably, the model’s performance in simulating the energy fluxes of LE, H, and R_n_ varied significantly across the different biomes and geographical latitudes but overall produced acceptable results in most cases.

The diurnal spatial distribution of LE and H fluxes revealed notably local differences across different biomes, suggesting that unique characteristics of each biome play an important role in shaping patterns of energy flux. With the inclusion of FVC and LAI, the model has shown that the distribution of fractional cover across biomes reflects the variation and diversity of vegetation, emphasizing the importance of heterogeneity in the distribution of heat fluxes. The partitioning of LE and H is also highly influenced by other factors beyond land cover. Previous SA studies on SimSphere have shown that apart from the fractional vegetation cover, the capability of the model to predict LE and H fluxes is significantly influenced by terrain factors, particularly aspect and slope. This is because both the quantity and angle of incoming solar radiation change in a diurnal circle, along with variations in shading from vegetation [44]. These factors, in turn, affect evapotranspiration by influencing LE and H fluxes. Beyond that, the model’s poor performance in rugged terrain conditions is largely attributed to the limited representativeness of local atmospheric conditions provided by the sounding data [37], especially in sites where the corresponding radiosondes were located far away (e.g., IT-CA2 and IT-CA3). The transfer of LE is also significantly affected by the surface moisture availability, as soil moisture supports evapotranspiration and thus controls the proportion of incoming solar radiation absorbed as latent heat [57]. Given that moisture availability in soil is important for accurately partitioning LE and H fluxes, site-specific representative values are also needed within the model’s parameterization [58].

R_n_ yielded the largest average error among all the simulated variables, which can be attributed to several factors. Overall, the model showed a consistent underestimation across most of the experimental sites, with an averaged RMSD and R^2^ values of 80.76 Wm^−2^ and 0.911, respectively. An important factor of influence of R_n_ distribution is the impact of topographical parameters, such as aspect and slope, which is expected, as slopes with varying orientations and angles receive different levels of solar radiation, controlling the distribution of solar and net radiation [59]. The intensity of solar radiation reaching the surface results from the interaction between topographical variations and vegetation characteristics [60,61]. This is also reflected in the representation of topographic characteristics in R_n_ spatial simulations (Figure 3c). Thus, in the case of this study, the sites in regions with gentle to significant slopes and intense topography are expected to perform with the lowest accuracy in the model simulations (e.g., IT-CA2, ES-Lju, and ES-Cnd). In addition to topographical features, previous research has pointed out the role of surface and foliage albedo in forming R_n_ distribution across different ecosystems, representing a complex interplay between topography and vegetation. The albedo, thus, affects the amount of R_n_ available for heat flux, including H and LE. This is important, as the partitioning of R_n_ into LE and H fluxes is known to be highly dependent on the vegetation and surface conditions of the site [12,62]. This is indicative of the need for accurate representation of surface and foliage albedo [63]. One of the primary determinants of R_n_ is land cover type, which affects both the albedo and rates of emissivity, as different land use types exhibit varying evapotranspiration rates, which are closely linked to the net radiation balance. Reference [64] noted that surface longwave radiation correlates with shortwave radiation, and adjustments to models can be made based on land cover and terrain, which directly affect surface albedo [64]. Additionally, the distribution of the R_n_ over soil and vegetation canopy is strongly affected by LAI, which is an important parameter for explaining both the R_n_ and all energy fluxes of the soil background such as LE and H. All these factors, including atmospheric conditions such as cloud cover and humidity, are significant in determining the way that solar radiation is absorbed, reflected, and emitted [65].

All in all, Sim*2D*Sphere effectively produced realistic estimates for the selected simulated parameters, particularly on cloud-free days and in flat-terrain regions. Although accuracy was limited for certain sites and dates, the model maintained a strong correlation between the predicted and observed energy flux values. The results highlight the influence of site-specific factors on model performance, particularly in terms of scatter contribution (Figure 3), which needs further investigation to identify the underlying causes of increased variability in certain biome and geographical locations.

#### Comparisons for Different Biomes

The model’s performance varied considerably across diverse biomes, particularly in simulating the energy flux of R_n_. The average statistics across all the biomes examined across all the days and sites are presented in Figure 5, where heat maps were used to display statistical metrics comparing the simulated and observed flux variables across different biomes. The grassland sites consistently showed higher model performance in comparison to all the other sites, with values indicating an excellent agreement with the observed diurnal evolution, followed closely by crop sites (and then forested areas, which demonstrated comparable performance between the ENF and DBF forest types. However, prediction accuracy was comparatively lower in the OSH and WSA sites located in the Subtropical Mediterranean climate zone in Spain, where the largest discrepancies were visible, especially for R_n_ flux (Figure 5). For LE fluxes, the lowest correlation between model predictions and in-situ data occurred in the shrubs and forested areas (ES-Lju, DE-Tha, and IT-CA3), while comparatively higher accuracy was obtained in the grassland (DE-Gri and FR-Tou) and croplands (FR-Aur, FR-Lam, DE-Kli, and IT-CA2) biomes. A similar pattern in terms of accuracy was also observed for H flux, with grasslands showing the lowest average errors, followed by croplands, while forested and shrub areas presented similar patterns of averaged RMSE. While the inclusion of diverse land cover types improved the model’s ability to simulate under different biomes, accurately parameterizing such complex environments is challenging and may impact model accuracy. In particular, forested areas with understory vegetation create a complex and heterogeneous environment that strongly influences energy and mass exchange. This complexity affects both the LE and H fluxes, as the intricate exchanges of mass and energy are shaped by ecosystem heterogeneity [66,67]. In addition, an important factor of influence in vegetated and forested areas is the canopy height, especially on H flux distribution, as plant shading reduces solar radiation on soil; decreases evaporation; and, particularly with trees, decelerates wind speed and increases transpiration [68]. Overall, the accuracy levels of the findings are comparable to those observed in prior validation studies conducted on Sim*2D*Sphere [43].

The results obtained herein are comparable to previous studies using the SimSphere 1D version of the model. The model’s performance for LE and H fluxes exhibited comparable statistical results on earlier versions of the SimSphere (1D) model in terms of both correlation accuracy and estimated errors [44,69]. On a site-by-site basis, the correlation results followed a trend of increased accuracy in the grassland and cropland sites, followed by the forested areas. Regarding LE flux, the same mean averaged errors were also obtained for certain biomes and geographical regions, consistent with the previous study in [69], where the DBF biome at a site in the wider region of Italy showed similar results (i.e., in the DBF biome of IT-CA3, a comparable RMSD of 64.68 was obtained, compared to an RMSD of 75.36 for IT-Col in the study in [69]). This trend was also observed for grassland sites, where the mean averaged errors were analogous. Overall, the averaged trend of LE errors is closely aligned with the model’s results reported in [69]. The study showed the same R^2^ value with [69], with a difference of −2.10 Wm^−2^ in the RMSD (averaged RMSD of 57.52 Wm^−2^ and an R^2^ value of 0.83). In the case of R_n_ flux, the average simulation accuracy reported in this study is lower in comparison to [43,69]. However, the averaged result of error for R_n_ is better compared to those reported in [66] (averaged RMSD value of 118.46 Wm^−2^). On a biome basis, the results showed a trend of increased accuracy in the grassland and cropland sites, followed by the forested areas, including woodland and shrubs. However, the model performance for R_n_ in the latter areas (forested) was not consistently higher compared to other biomes, as observed in [69].

The results, particularly those of the LE and H fluxes, were also comparable to those from other land surface models, where the highest accuracy was achieved in sites under herbaceous vegetation with the model’s performance being notably lower in forested and shrubland areas [68]. For example, [70] found similarly poor performance when analyzing shrublands using tower-based data, which mainly attributed this outcome to challenges in model parameterization over such landscapes, which are characterized by significant heterogeneity and inherent water limitations. In this regard, better simulations of energy fluxes were obtained for grasslands and cropland sites where shorter canopies dominate, rather than forested, shrub, or woodland savanna ecosystems [20,56,68]. In terms of block performance, the variability in simulation accuracies for energy fluxes under the crop’s biome in block sites of France suggests that certain localized factors, such as soil physiological properties and management practices, may be contributing to the higher scatter observed at specific crop sites. These regional variations in model performance highlight the site-specific nature of the results, suggesting that localized factors such as vegetation and physical soil properties might be influencing the accuracy of the model predictions. This means that when different biomes are simulated simultaneously, more detailed and site-specific parameters must be taken into account, particularly due to their similar responses and diurnal variations in flux exchanges during heat waves or dry–wet environmental conditions. This is especially important due to the fact that soil moisture and thermal inertia play a significant role in influencing these exchanges.

The differences observed between this study and previous research using SimSphere –primarily focused on the model’s 1D version– suggest that the model’s performance is influenced by the parameterization process, which was designed for a single, specific PFT per simulation rather than accounting for multiple biomes simultaneously. In this context, potential differences may arise if the average values that were set across different biomes in a block of sites fail to capture local variations. Although the model simulated the diurnal variability of flux with adequate accuracy across the selected biomes, the need for further refinement in model calibration is indicative as confirmed by the limitation of Sim*2D*Sphere to represent the exchange of fluxes, especially in certain biomes such as shrubs and forested areas is evident, during different seasons. In general, almost all models exhibit lower performance when applied to forest ecosystems but show better results over areas with shorter vegetation. As McCabe noted, this is likely attributed to the basic physical principles behind models like Penman–Monteith, Priestley–Taylor, and Monin–Obukhov flux gradient functions, which were designed specifically for these types of surfaces [68]. Overall, the model’s simulations provided realistic estimates of the diurnal spatiotemporal variability of the parameters simulated by the model, which are in agreement with land cover distribution and topographical features of the areas.

### 5.2. Potential Sources of Uncertainty and Limitations

Detailed information on meteorological forcing data and geophysical parameters is of critical importance in the parameterization process. A main constraint in the current study is that since exclusively EO data were not used to force these parameters in the current simulations, the model applied a uniform parameterization value for the other key input values, such as surface and foliage albedo, soil moisture-related physiological properties, canopy height, and surface roughness, across all the biomes within each simulated block. The current approach, while simplifying the parameterization process, could have limited the model’s ability to capture unique local conditions, potentially introducing bias. Thus, significant discrepancies between the simulated and observed datasets could be a result of the selected model’s parameterization. As [71] stated, using more generalized parameters reduces the accuracy of fluxes, as the selection of parameter classifications significantly influences the accuracy of simulated photosynthesis and transpiration fluxes [65]. Hence, to effectively assess the spatial variability of simulated energy fluxes across diverse biomes with higher accuracy, incorporating exclusively EO data into the parameterization process is a straightforward approach for capturing local variations in soil, vegetation, and atmosphere behavior.

However, another important aspect to consider is whether the model input values obtained from EO datasets differ from the site measurement due to differences in spatial scale. The spatial scaling is recognized as a challenging issue, particularly in surface-atmosphere exchange affected by heterogeneities in vegetation properties, topography, and texture of the soil. Since energy and heat fluxes are highly influenced by land surface heterogeneity, the variability in land cover within a pixel or model grid size can result in significant errors in the mean pixel or grid heat flux estimation [72]. To this end, such discrepancies may still arise due to the difference in spatial resolution between EO data and the flux footprint. According to the model’s extensive sensitivity analysis studies [41], the most influential factors for the parameters under investigation are topographical features, such as slope and aspect. The SRTM DEM, which was used to derive these parameters, may introduce some uncertainty regarding how accurately the topography is represented at the required level of detail. Uncertainty in the current flux predictions can also be found in the EO data used to generate FVC and LAI indices for the parameterization process may have introduced certain biases. This bias is particularly evident in sparse vegetation, where detection limits and potential saturation effects may result in an average bias of up to 20% [55,73]. While some models show limited sensitivity to LAI bias, flux predictions are highly sensitive to canopy height estimates derived from LAI, especially when the canopy height is close to the height of micrometeorological measurements [20]. In this context, the CLC 2018 that was used to define the land cover types in the model’s input parameters may introduce uncertainties, as due to spatial resolution (250 m), it does not support sufficiently the parameterization of PFTs given the evident heterogeneity of European ecosystems and particularly in forested regions, although efforts were made to minimize these effects by selecting regions that are as homogeneous as possible.

Other potential sources of uncertainty include unusual diurnal flux that varies significantly, thus creating spike variations (as illustrated by the LE spikes observed on a summer day in June in Germany in Figure 6a). These variations may result from the presence of strong advective or convective conditions due to surface heterogeneities or non-stationary turbulence conditions [43,74,75]. Other potential sources include systematic measurement inaccuracies caused by discrepancies in the observation of footprints. Instrumentation errors in measurements of flux tower, evidenced by extreme spikes, impact data quality and hence, induce errors in metrics. According to [76], the accuracy error for R_n_ in-situ is typically about 10%, added to an additional 10% uncertainty due to the limitations of instrumentation due to viewing angle and measuring volume, particularly in rugged terrains. Additionally, EC tower measurements often face an energy balance closure issue, with imbalances ranging from 10% to 30%, particularly evident in half-hourly averages [34]. These imbalances cannot be fully captured or replicated by SimSphere, as the model inherently assumes a closed energy balance in its simulations.

### 5.3. Computational Requirements and Model Transferability

Sim*2D*Sphere requires consistent geospatial data inputs, including the same coverage areas and size of pixels, across all the parameters that are allowed to be driven by geospatial input. The high computational demands of simulations increase the time required to run the simulation. For example, running simulations with a 30 × 30 km^2^ Landsat image with a 30 m pixel size took approximately 9h on a computer with 16 GB of RAM and an x64-based processor. However, this issue can be partly addressed through the integration of High-Performance Computing (HPC) with cloud-based services, along with the fact that many space agencies provide EO datasets and operational products through cloud systems and open access servers.

In terms of generality, ref. [44] stated that the integration of detailed land cover information in the SimSphere parameterization process can provide more insights into the model’s performance. Expanding the land cover types within the input geospatial data would help refine the model’s performance, especially in complex forested areas. To this end, the model’s generality and transferability has been improved by incorporating more PFT’s, which would provide a more comprehensive assessment of its applicability across diverse land cover types, particularly heterogeneous forests where simulation accuracy often decreases.

The model produced realistic simulations of energy fluxes that corresponded well with the tested environmental conditions, underscoring its generality. Yet, further parameter tuning is necessary to adjust the model’s parameterization for specific applications, the model as proven by the statistical analysis results obtained herein satisfies the standards of accuracy, realism, and generality according to [76].

## 6. Final Remarks

The current research evaluated, for the first time, the ability of the Sim*2D*Sphere model to estimate LSIs across European ecosystems. The recently developed modified version of SimSphere model, namely Sim*2D*Sphere, allowed the integration of EO data into its parameterization process, thereby providing detailed spatiotemporal variability of LSIs parameters at unprecedented spatial and temporal resolutions. Sim*2D*Sphere’s capacity to predict the key parameters associated with LSIs was tested for the first time across six European ecosystems. The experimental set-up was designed to simulate energy and water fluxes, on a diurnal basis (06:00–23:30), at a 30 min time interval. To validate the model’s predictions of LE, H, and R_n_, data were collected from ten FLUXNET towers in the European continent (3 days per block of sites) covering different years, primarily during the summer season (simulation days range from June, July and August) and early autumn (September).

Overall, the highest simulation accuracy was achieved in grassland and cropland sites, while performance was notably lower in forested areas, WSA, and OSH. Sim*2D*Sphere for most of the days in which it was evaluated successfully identified the expected diurnal patterns of energy and water fluxes, even if not always their exact magnitudes, particularly for cloud-free days and sites with flat terrain at sites under grassland biome. The accuracy achieved is in agreement with similar model validation studies to the previous SimSphere model (1D) version and is comparable to the preliminary results of the current modified version conducted under varied conditions, as well as to other models in terms of predictive accuracy over those types of biomes.

All in all, the study suggests that the Sim*2D*Sphere SVAT model can effectively be combined with EO data in different spatial scales to simulate the variability of LSIs and provide spatially explicit temporal maps of flux local variations, even without the excessive requirement for tuning its parameters to specific site’s characteristics. Yet, in terms of the model’s performance, the results slightly underscore areas for improvement across different ecosystems and seasonal cycles. Further testing of the model in diverse biomes and under varying conditions, including different seasons, topographical features, and climate zones, is required. Additionally, further adjustments are necessary to refine the model’s parameterization, including plant and soil physiological properties, to improve representations of the spatial variability of energy and water fluxes under different biomes. The implementation of site-specific parameters would definitely improve the performance of the model. As a broader parameterization scheme is applied, the discrepancies and regional variations between sites increase. To conclude, expanding the 2D model verification using also a variety of EO datasets into the parameterization process, particularly those at higher resolutions, and assessing its performance under different conditions, represents a key direction for future development in accurately simulating LSIs exchanges. The findings of this study pave the way for broader implementation of the Sim*2D*Sphere model, with expected advancements from incorporating satellite data to more accurately capture water-limited fluxes.

## Figures and Tables

**Table 1:** List of eddy covariance sites used in the study and their characteristics. The biomes are defined according to the International Geosphere–Biosphere Programme (IGBP), which includes the following biomes studied: open shrublands (OSH), woody savannas (WSA), croplands (Crop), grasslands (GRA), evergreen needleleaf forests (ENF), and deciduous broadleaf forests (DBF). The Köppen classification refers to the Köppen climate system, with Temperate Oceanic Climate (Csb) and Hot-Summer Mediterranean Climate (Csa) identified in the study sites.

Dates	Site	Lat/Lon	Height (~ in m)	Slope	Biome	Plant Type	Soil Type	Köppen
11 June 2020 29 July 2020 14 August 2020	ES-LJU	36.926/2.752	2.5	Gentle (<2%)	OSH	evergreen shrubs (macchia)	Clay loam	Csa
	ES-CND	37.915/−3.227	15	Medium (>2%, <5%)	WSA	Olive cultivars	Clay loam	Csa
27 June 2019 29 July 2019 30 August 2019	FR-AUR	43.549/1.106	3.5	Significant (>5%, <10%)	CROP	wheat	Clay loam	Cfb
	FR-LAM	43.493/1.237	3.6	Flat	CROP	maize	Clay loam	Cfb
	FR-TOU	43.572/1.374	3	Flat	GRA	Grass	Clay-loam	Cfb
30 June 2018 10 August 2018 18 September 2018	DE-KLI	50.892/13.522	2	Flat	CROP	maize	Loam	Cfb
	DE-GRI	50.950/13.513	3	Flat	GRA	C3 short grass	Loam	Cfb
	DE-THA	50.964/13.567	42	Gentle (<2%)	ENF	evergreen coniferous trees (Norway Spruce)	Loam	Cfb
27 July 2013 12 August 2013 13 September 2013	IT-CA2	42.377/12.026	5	Gentle (<2%)	CRO	crop rotation grassland	Clay loam	Csa
	IT-CA3	42.380/12.022	5	Flat	DBF	temp. BL deciduous trees	Clay loam	Csa

**Table 2:** Statistical measures used to assess the agreement between the predictions and ground observations. Subscripts *i* = 1… *N* refer to the number of individual observations, while *O* and *P* refer to the observed and predicted values.

Name	Description	Mathematical Definition
Bias/MBE	Bias (accuracy) or Mean Bias Error	$bias=MBE=\frac{1}{N}\overset{N}{\underset{i=1}{\sum }}\left(P_{i}-O_{i}\right)$
Scatter/MSD	Scatter (precision) or Standard Deviation	$scatter\left(\sigma \right)=\sqrt{\frac{1}{\left(N\right)}\overset{N}{\underset{i=1}{\sum }}(P_{i}-O_{i}-\bar{\left(P_{i}-O_{i}\right)}{)}^{2}}$
RMSD	Root Mean Square Difference	$RMSD=\sqrt{bias^{2}+scatter^{2}}$
R^2^	Linear Correlation Coefficient	$R^{2}=\frac{{\sigma^{2}}_{xy}}{\sigma_{x}^{2}\sigma_{y}^{2}}=\frac{{[n\sum xy-(\sum x)(\sum y)]}^{2}}{[n(\sum x^{2})-(\sum x{)}^{2}][n(\sum y^{2})-(\sum y{)}^{2}]}$

**Table 3:** Average diurnal simulation accuracy for each simulated parameter across all the simulated days. Bias, scatter, and RMSD are expressed in Wm^−2^. The *n* refers to the sample size of in-situ measurements. The symbols with an upper bar represent mean values.

Country	Station	Parameter	Bias	Scatter	RMSD	R^2^	*n*
Spain	ES-Lju	Latent heat flux (Wm^−2^)	38.87	43.94	58.66	0.250	108
	ES-Cnd		−8.15	30.13	31.22	0.634	108
France	FR-Aur		44.14	49.29	66.16	0.778	108
	FR-Lam		−23.42	54.66	59.47	0.805	108
	FR-Tou		21.10	39.25	44.56	0.826	108
Germany	DE-Kli		7.53	28.31	29.30	0.890	108
	DE-Gri		39.79	42.65	58.33	0.748	108
	DE-Tha		39.16	44.53	59.30	0.465	108
Italy	IT-CA2		39.37	52.09	65.29	0.584	108
	IT-CA3		−22.39	60.68	64.68	0.645	108
**Average**			**17.6**	**44.53**	**53.69**	**0.662**	
**Country**	**Station**	**Parameter**	**Bias**	**Scatter**	**RMSD**	**R^2^**	***n***
Spain	ES-Lju	Sensible heat flux (Wm^−2^)	0.38	58.96	58.96	0.792	108
	ES-Cnd		26.84	56.88	62.90	0.802	108
France	FR-Aur		−4.24	38.33	38.57	0.945	108
	FR-Lam		79.43	59.14	99.03	0.812	108
	FR-Tou		9.66	39.99	41.14	0.886	108
Germany	DE-Kli		8.53	36.96	37.93	0.861	108
	DE-Gri		27.22	34.39	43.86	0.891	108
	DE-Tha		−30.57	58.78	66.26	0.928	108
Italy	IT-CA2		−20.44	61.07	64.40	0.744	108
	IT-CA3		29.16	54.94	62.20	0.731	108
**Average**			**12.59**	**49.94**	**57.52**	**0.839**	
**Country**	**Station**	**Parameter**	**Bias**	**Scatter**	**RMSD**	**R^2^**	***n***
Spain	ES-Lju	Net Radiation (Wm^−2^)	−31.54	130.17	140.23	0.788	101
	ES-Cnd		−22.97	110.98	113.33	0.828	108
France	FR-Aur		3.185	48.63	48.73	0.964	108
	FR-Lam		−29.92	73.17	79.05	0.929	108
	FR-Tou		−15.51	74.19	75.80	0.921	108
Germany	DE-Kli		−0.699	65.74	65.75	0.934	108
	DE-Gri		15.04	68.01	69.65	0.934	108
	DE-Tha		−37.55	83.14	91.23	0.943	108
Italy	IT-CA2		−4.00	58.80	58.94	0.937	106
	IT-CA3		−9.21	64.28	64.93	0.934	107
**Average**			**−13.31**	**77.71**	**80.76**	**0.911**	

## Data Availability

Data will be made available upon request.
